# The “supercompensation” effect of children’s lockdown during COVID-19: based on the analysis of changes in physical activity, sleep, and psychology

**DOI:** 10.1186/s12889-024-19035-2

**Published:** 2024-06-06

**Authors:** Yunong Li, Guanggao Zhao, Liqiang Su, Jinmei Fu, Shunli Sun, Ruiming Chen, Delong Chen, Xuewen Hu, Tianle Jiang, Fanchao Shen

**Affiliations:** 1 School of Physical Education, Nanchang University, Nanchang, 330031 China; 2 School of Physical Education, Jiangxi Normal University, Nanchang, 330027 China; 3 Jiangxi Sports Science Medicine Centre, Nanchang, 330006 China

**Keywords:** Preschooler, Lockdown, Physical activity, Psychological, Sleep, Supercompensation

## Abstract

**Objective:**

To investigate the “supercompensation” effect of preschoolers during the coronavirus disease 2019 lockdown by comparing the changes in physical activity (PA), psychological, and sleep indicators before and after the lockdown.

**Methods:**

A total of 127 children (aged 3–6 years) were recruited. Before and after the lockdown, the children’s PA levels were measured using the ActiGraph GT3X^+^, and their psychological and sleep indicators were measured using the Strengths and Difficulties Questionnaire (SDQ) and Child Sleep Habit Questionnaire (CSHQ), respectively.

**Results:**

Regarding PA, the children’s total physical activity, low-intensity physical activity, and medium-intensity physical activity (MVPA) were higher after the lockdown than before the lockdown, with significant differences in MVPA (*p* < 0.05). Regarding psychology, the children’s SDQ and multidimensional scores were better after the lockdown than before the lockdown, with a significant difference in SDQ scores (*p* < 0.05). Regarding sleep, the children’s CSHQ scores were better after the lockdown than before the lockdown, with a highly significant difference in CSHQ scores (*p* < 0.01).

**Conclusion:**

After lockdown, children’s PA, psychological, and sleep effects were “supercompensated.” In particular, the PA of preschoolers before, during, and after the lockdown may show a “baseline-inhibition-supercompensation” process.

The outbreak of the coronavirus disease 2019 (COVID-19) poses a serious threat to the lives and health of people worldwide. Faced with the serious situation of the epidemic, governments and international organizations had to take measures to reduce the effect of the epidemic. According to the World Health Organization (WHO), lockdown can effectively reduce the incidence of COVID-19 and deaths [1]. Since the end of 2022, the epidemic situation in China has been generally stable [2], and on May 5, 2023, the WHO declared an end to the COVID-19 global emergency. However, COVID-19 and Influenza A virus outbreaks are still sporadic in some areas, and the routine measures for epidemic prevention and control have shifted from being centralized and instituting lockdown measures to voluntary home isolation by residents.

While lockdown is effective in protecting human life and health, it may still affect physical and mental health to some extent. Some studies have found that people’s physical activity (PA) levels were significantly reduced during the lockdown [3–5], which led to psychological and sleep problems [6–8]. For preschoolers in the early stages of life, their healthy physical and mental development is the foundation of the life cycle [9], and the negative effects of these problems may be profound [10–12]. National and international studies have shown that children’s PA and mental health levels are significantly lower during lockdown than before lockdown [13–16], but less studies have been conducted to characterize changes in children’s health after lockdown.

To ensure that the physical and mental health of preschoolers is preserved during the COVID-19 epidemic, our group organized a large-scale PA and physical health evaluation service for preschoolers for 2 consecutive years. Interviews of kindergarten teachers and parents of children revealed that during this period, some children who experienced lockdown tended to perform better in health behaviors (such as PA) after lockdown compared with before lockdown. Our group hypothesized that lockdown during the epidemic may stimulate a “supercompensation” effect similar to that of exercise training. Supercompensation, also known as “overcompensation” or “super recovery,” refers to the process of rebuilding body structure and function after training such that physical function is not only restored but also exceeded [17–19]. In 2022, Hurter et al. found that PA levels in children and adolescents (aged 8–18 years) were significantly higher after lockdown than during lockdown [20], but it is unknown whether the “supercompensation” effect was further increased beyond pre-lockdown levels, and no studies have been reported on the preschooler population.

In the latter part of 2022, after the first phase measurements of PA and psychological and sleep health indicators of preschoolers, the COVID-19 broke out in the area where the assessed preschoolers were located. Our group was quarantined in a designated hotel while the children were quarantined at home. After the lockdown, our group completed the measurements of some of the health indicators, with the approval of the local government, kindergarten students, and parents, aiming to explore the possible “supercompensation” effect of preschoolers’ lockdown to contribute to the improvement of lockdown measures and the health of preschoolers after an epidemic or other major public events. This study aimed to provide basis for the improvement of lockdown measures and implementation of health promotion for preschoolers after public health events.

## Subjects and methods

### Object of study

Based on the “Physical activity measurement and physical health evaluation of preschoolers in Jiangxi Province” study (for details, please refer to the document 赣體科会字[2022]4号), 239 healthy children aged 3–6 were recruited from three kindergartens in Pingxiang City, Jiangxi Province. The three kindergartens are separately located in the city, county, and village, respectively. The 127 children, including 59 boys and 68 girls, who completed all the tests and had complete and valid data before and after lockdown, were included as participants for this study (Table 1).

**Table 1:** Basic information of participants

Basic features	Male (*n* = 59)	Female (*n* = 68)	Total (*n* = 127)
Age (year)	4.83 ± 0.89	4.56 ± 0.98	4.69 ± 0.95
Height (cm)	115.00 ± 6.49	107.06 ± 12.26	108.38 ± 11.40
Body weight (kg)	20.10 ± 5.37	18.50 ± 2.39	19.83 ± 4.84
BMI (kg/m^2^)	14.90 ± 1.91	14.85 ± 1.10	14.88 ± 1.58

All the participants had parental consent to participate in this study and signed the informed consent form. During the testing process, researchers contacted parents via WeChat, and the children and parents could withdraw from the study and withdraw their consent at any point in the study. The study protocol was approved by the Medical Research Ethics Committee of The Second Affiliated Hospital of Nanchang University (approval number: 研临审 [2020] No.125).

### Test indicators and methods

The lockdown period for preschoolers was from November 5 to December 7, 2022, and PA, psychological, and sleep tests were conducted 7 to 14 days before and after lockdown. Parents filled an online questionnaire to provide basic information about their child (including the child’s name, class, age, height, weight, and other basic demographics). All the tests were presented to the outbreak prevention and control department for approval and were completed after permission was granted by the Education Department and kindergarten authorities.

#### Physical activity test

The PA level was tested using a tri-axial accelerometer, ActiGraph GT3X^+^, for 7 consecutive full days (5 school days + 2 rest days) before and after lockdown. A micro-group of parents and researchers was set up prior to the test to remind parents daily to ensure that their children wore the device and to answer questions. The parents assisted their children in putting on and removing the device during the test. During school day, the researcher checked the child’s device once in the morning and in the evening and made adjustments. Using WeChat, the researchers reminded parents every day on the weekend to help their children wear the accelerometer and ensure they do not remove it. The accelerometers were collected at the end of the test, and the data was downloaded and processed using ActiLife (Version 6.13.4). All parents whose children participated in the test received a PA report after the test, including 7 days of total physical activity (TPA), light physical activity (LPA), moderate to vigorous physical activity (MVPA), sedentary behavior (SB), step counts, and whether the WHO recommended amount was met.

The ActiGraph GT3X^+^ measurement parameters were set with reference to the relevant literature [21, 22]. The parameters were set using the Butte cut-point [23]. The metrics included LPA, MVPA, and SB. TPA is the sum of LPA and MVPA (Table 2). According to the Chinese Guidelines for Physical Activity in Children and Adolescents [24], children with MVPA > 60 min/day were considered as qualified and children with MVPA < 60 min/day were considered as unqualified. Considering that children’s activities are limited during the regular control period, the average MVPA value after lockdown has been reported as 50.8 min/day in existing studies [25]. Therefore, this study reduced the Chinese guideline standard by 10 min/day and used 50 min/day as the threshold for further comparative analysis.

**Table 2:** ActiGraph GT3X+ test method and parameter setting

Number	Contents		Requirements
1	Wearing area		Fixed to the upper right iliac crest
2	Sampling interval		15 s
3	Unworn time definition		Choi Algorithm [26]
4	Test sample inclusion criteria		≥3days (2 school days+1 rest day), ≥480 min/day
5	Intensity Boundary	SB	Counts ≤239/60 s
		LPA	240/60 s≤Counts≤2119/60 s
		MVPA	Counts ≥2120/60 s

#### Psychological test

The parent version of the SDQ, compiled by the American psychologist, Goodman, is used to assess early childhood mental health [27]. An online questionnaire was administered to parents of preschoolers during the PA test, and parents were asked to respond based on their child’s recent mental health performance. The questionnaire consists of 25 items, divided into five dimensions: emotional symptoms, conduct problems, hyperactivity, peer interaction problems, and pro-social behavior, each with 5 questions. The questionnaire was scored on a three-level scale of 0, 1, and 2 for “does not meet,” “somewhat meets,” and “fully meets,” with reverse scoring for negative items.

The total score for the questionnaire is the cumulative score for all items. The total score for difficulties is the cumulative score for the four dimensions of emotional symptoms, conduct problems, hyperactivity, and peer interaction problems, with higher scores indicating more serious psychological problems. Pro-sociality is a measure of pro-social behavior, with lower scores indicating more serious pro-social behavioral problems. According to the criteria provided in the questionnaire, a total difficulty score of ≤ 13 is considered “normal,” 13 < total difficulty score of < 17 is considered “marginal,” and a total difficulty score of > 17 is considered “abnormal.”

#### Sleep test

The CSHQ, developed by Judith, Professor of Pediatrics at Brown University, was used to test the sleep habits of young children [28]. An online questionnaire was administered to parents of preschoolers during the PA test, parents were asked to respond based on their child’s recent sleep habits. The questionnaire contains 33 scored questions, divided into five dimensions: sleep habits, sleep behavior, night waking, morning wakefulness, and daytime sleepiness, with each dimension containing 2–15 questions. The questionnaire is scored on a three-level scale of “occasionally,” “sometimes,” and “usually,” with scores of 1, 2, and 3, respectively, and reverse scoring for negative items.

The questionnaire scores are cumulative scores for the items included, with higher scores indicating more serious sleep problems. A score of < 41 is considered “normal” and a score of > 41 is considered “abnormal”.

### Statistical analysis

Data were processed using SPSS 26.0. Data that conformed to a normal distribution were described using mean ± standard deviation, and those that did not conform to a normal distribution were described using the median (interquartile spacing). Paired samples t-test, Mann- Whitney U-test, and χ^[2]^ test were used to compare the differences between the indicators before and after the lockdown. The level of significant difference was set at *p* < 0.05 and of very significant difference was set at *P* < 0.01.

## Study results

### Physical activity indicators

Of the 127 children recruited for this study, 81 (males, 40 and females, 41) participated in the PA test, and the data were complete and valid. Statistical analysis showed that SB levels were lower after than before lockdown and LPA, MVPA, and TPA levels were higher after than before lockdown, with significant differences in MVPA levels (*p* < 0.05; Table 3).

**Table 3:** Changes in the SB, LPA, MVPA, TPA levels of preschoolers before and after lockdown

PA type	Before lockdown	After lockdown
SB	864.16 ± 368.80	793.43 ± 310.15
LPA	100.41 ± 19.60	102.18 ± 15.02
MVPA	56.09 ± 15.17	59.96 ± 13.69*
TPA	155.50 ± 34.41	162.24 ± 28.73

“*” indicates *P* < 0.05 compared with before lockdown

**Fig. 1:**
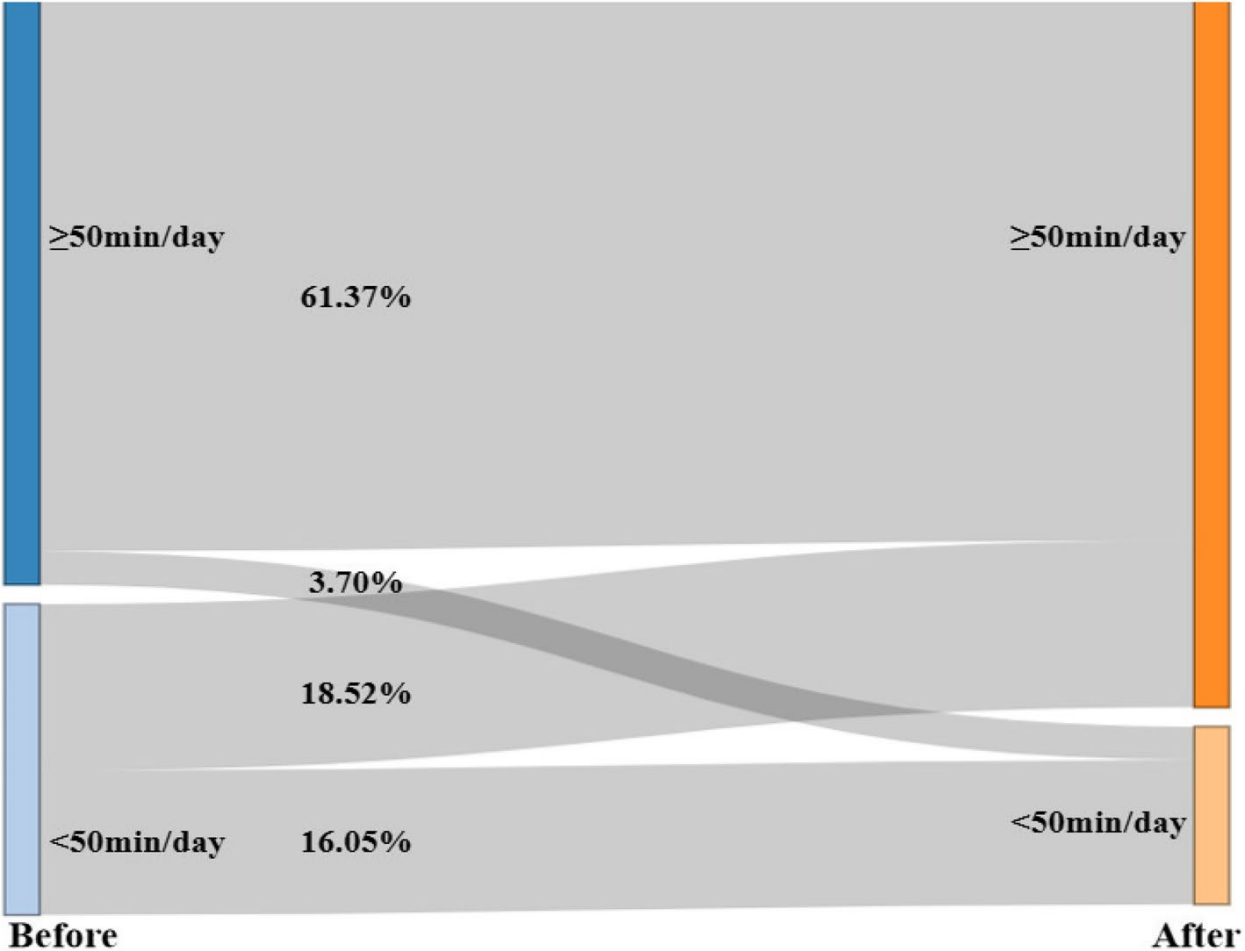
Sankey diagram of preschoolers’ MVPA before and after lockdown

The rate of those who had qualified MVPA levels was higher after lockdown (48.15%) than before lockdown (45.68%), with 20.99% of children moving from unqualified to qualified and 18.52% moving from qualified to unqualified. Using 50 min/day as the cut-off, the Sankey diagram results showed that the proportion of children with MVPA levels > 50 min/day was significantly higher after lockdown (80.25%) than before lockdown (65.43%) (*p* < 0.01), with 18.52% of children having a change in MVPA levels from < 50 min/day to ≥ 50 min/day and 3.70% moving from ≥ 50 min/day to < 50 min/day (Fig. 1).

### Psychological indicators

Of the 127 children recruited for this study, 60 (26 males and 34 females) participated in the psychological test, and the data were complete and valid. Statistical analysis showed that the total SDQ scores and each dimension scores were lower after lockdown than before lockdown, except the behavioral problems dimension, for which a significant difference was observed between the total scores and change in pro-sociality (*p* < 0.05; Table 4).

**Table 4:** Comparison of preschoolers’ SDQ scores before and after lockdown

Dimensionality	Before lockdown	After lockdown
Total Score	18.50 ± 3.87	17.60 ± 4.54*
Hardship Score	11.49 ± 3.44	11.06 ± 3.78
Emotional Symptoms	1.71 ± 1.73	1.52 ± 1.55
Character Issue	1.71 ± 1.07	1.77 ± 1.21
More Movement	3.83 ± 1.40	3.60 ± 1.27
Peer Interaction	4.31 ± 1.11	4.23 ± 1.13
Pro-sociality	6.94 ± 2.01	6.48 ± 2.28*

“*” indicates *p* < 0.05 compared with before lockdown

**Fig. 2:**
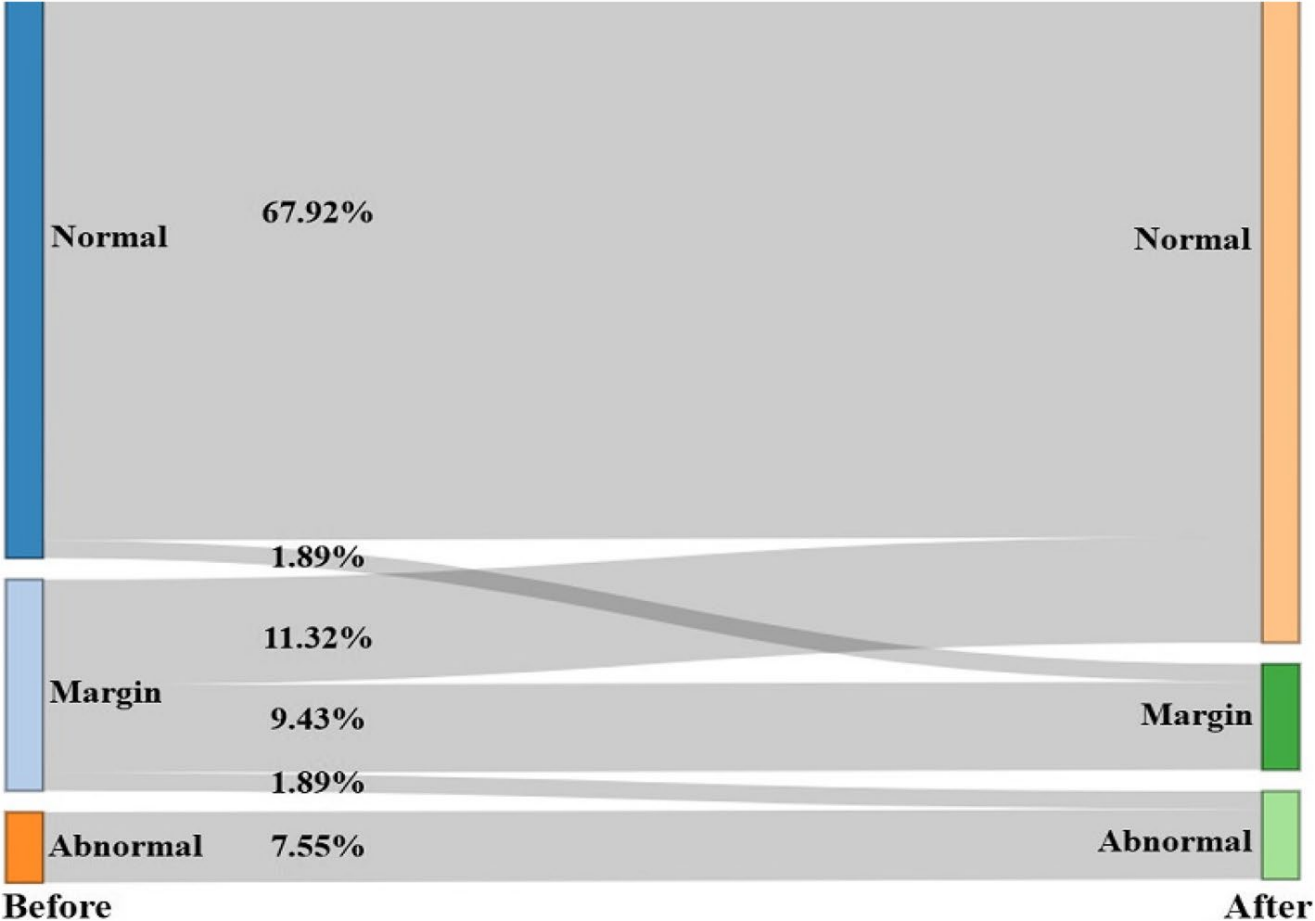
Sankey diagram of preschoolers’ SDQ score rates before and after lockdown

The results of the Sankey diagram showed that the detection rate of psychological normality was higher after lockdown (79.24%) than before lockdown (69.81%) (*p* > 0.05), with 11.32% of the children moving from margin to normal, 1.89% moving from normal to margin, and 1.89% moving from margin to abnormal (Fig. 2).

### Sleep indicators

Of the 127 preschoolers recruited for this study, 60 (26 males and 34 females) participated in the sleep index test, and the data were complete and valid. Statistical analysis showed that the total score and all the various dimension scores were lower after lockdown than before lockdown, with a highly significant difference (*p* < 0.01) in the total score and the morning rise dimension score (Table 5).

**Table 5:** Comparison of preschoolers’ CSHQ scores before and after lockdown

Dimensionality	Before lockdown	After lockdown
Total Score	46.55 ± 5.64	43.24 ± 5.96**
Bedtime Habits	14.16 ± 2.74	13.76 ± 2.64
Sleeping Behavior	18.71 ± 2.58	18.24 ± 3.00
Wake up at Night	2.45 ± 0.86	2.43 ± 0.78
Morning Raising	7.75 ± 1.90	6.39 ± 1.34**
Daytime Sleeping	2.41 ± 1.22	2.41 ± 1.39

“**” indicates *p* < 0.01 compared with before lockdown

**Fig. 3:**
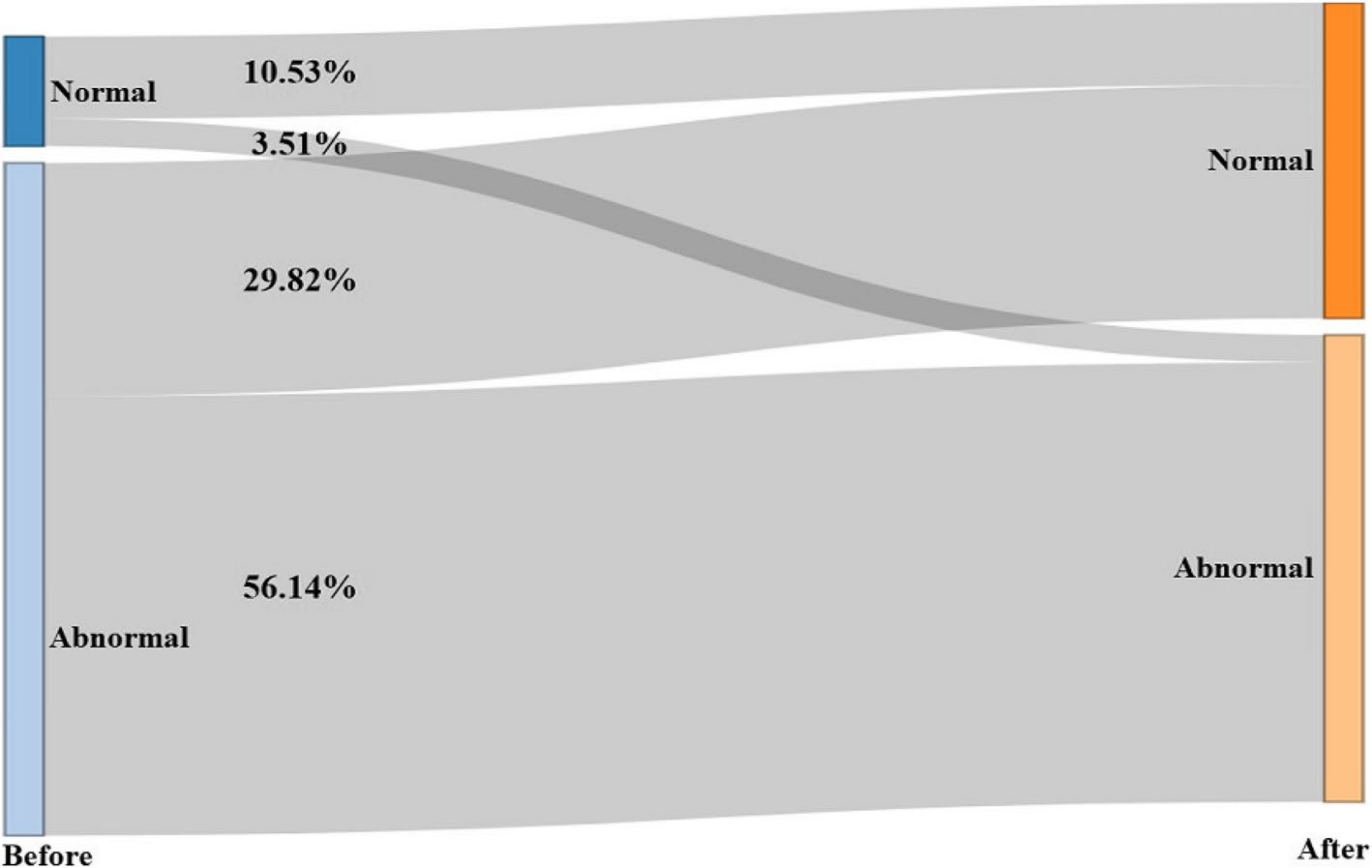
Sankey diagram of preschoolers’ CSHQ score rates before and after lockdown

The Sankey diagram shows that the detection rate of normal sleep was significantly higher (40.35%) after lockdown than before lockdown (14.04%) (*p* < 0.01), with 29.82% of preschoolers changing from abnormal to normal and 3.51% changing from normal to abnormal (Fig. 3).

## Discussion

Since the end of 2022, the global epidemic situation has been generally stable, while the COVID-19 and other epidemics are still sporadically spread in local areas. In normalizing home isolation, research on the physical and mental health of various populations, especially young children in the early years of life, is of great importance to the future of a country and the development of human society. In an article published in *Lancet Psychiatry*, the COVID-19 pandemic has been reported to have a long-term impact on the well-being of children, and academics need to quickly collect high-quality data to determine the impact of lockdown on children [29]. This study is the first known study on the characteristics of physical and psychological indicators of preschoolers before and after lockdown and to investigate the possible “supercompensation” effect of lockdown as a reference for governments, education authorities, and families of preschoolers.

### Physical activity

**Fig. 4:**
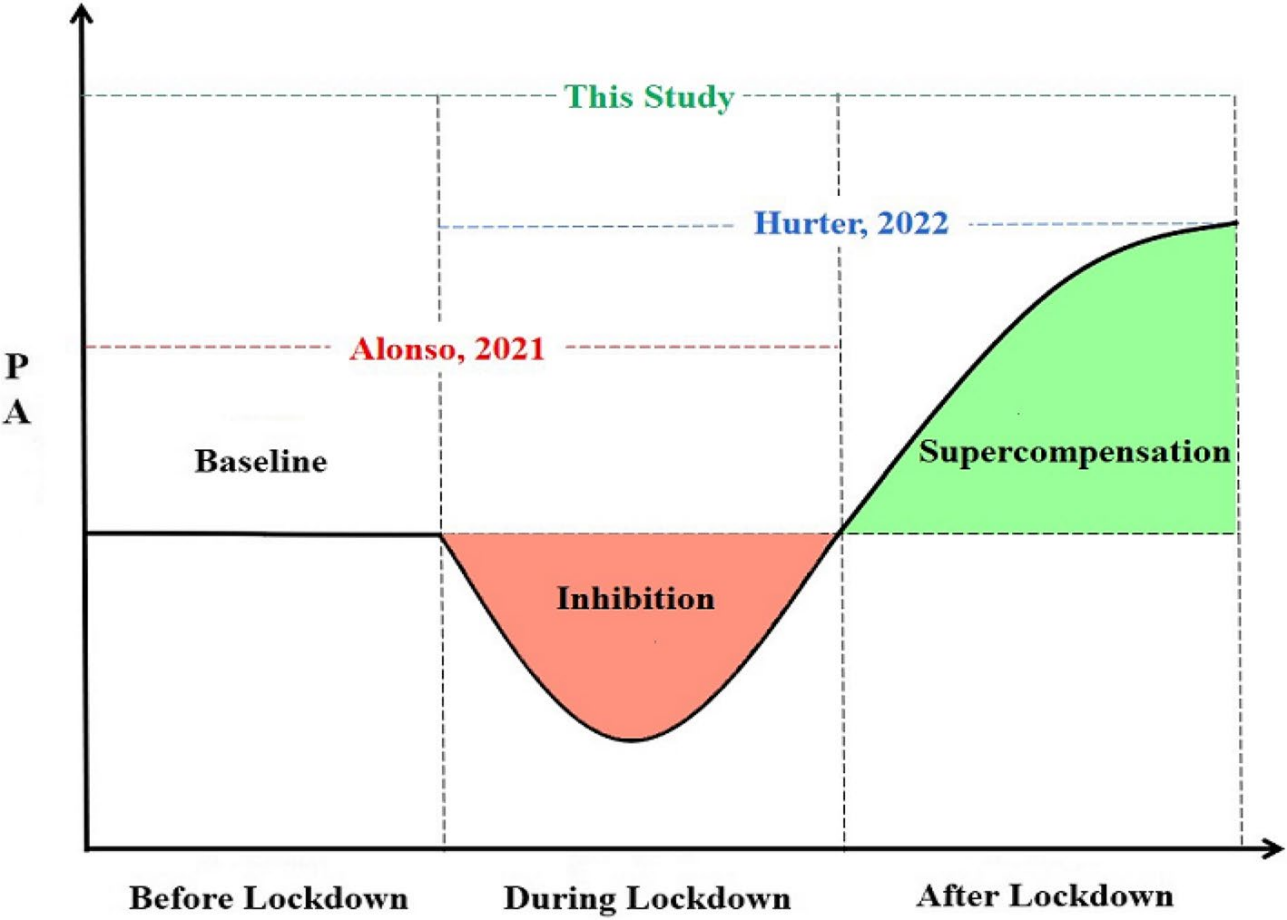
The “supercompensation” of preschoolers’ physical activity before, during, and after lockdown

This study found that LPA, MVPA, and TPA levels were higher after lockdown than before lockdown, with significant differences in MVPA levels (*P* < 0.05); the rate of preschoolers meeting the MVPA standard after lockdown (48.15%) was higher than before lockdown (45.68%), while the proportion of preschoolers with MVPA ≥ 50 min/day was significantly higher after lockdown (80.25%) than before lockdown (65.43%) (*P* < 0.01), suggesting that a “supercompensation” effect may have occurred in PA levels, especially MVPA, after lockdown. Previous studies have investigated the changes in PA in children before, during, and after lockdown. Alonso et al. (2021) found that TPA and MVPA levels were significantly lower (*P* < 0.05) during lockdown than before lockdown [25]. Hurter et al. (2022) found that TPA and MVPA levels were significantly higher (*P* < 0.01) after lockdown than during lockdown [20]. Based on this study and previous research, the “baseline-inhibition-supercompensation” process of PA in young children before, during, and after lockdown has been fully outlined (Fig. 4).

Since the famous psychologist, Alfred Adler, first introduced compensation in 1907, the term has been widely used in the fields of psychology and physiology. In 1941, Yampolyskaya and others proposed the Supercompensation theory based on sports training practice, and it has been accepted in sports science worldwide [30]. This study suggests that the “baseline-inhibition-supercompensation” changes in PA in children before, during and after lockdown may be related to differences between the kindergarten and home environments. During lockdown, children’s range of activities reduced, and activities such as peer interaction were affected [31]. The PA activities and peer-to-peer play children receive in the kindergarten cannot be replaced by online classes [32, 33]. Therefore, children’s opportunities to participate in PA were reduced, and their PA changed from normal to suppressed. However, after a short period of lockdown, children did not lose their PA habits, and with their active nature and appropriate parental guidance, their motivation to participate in PA increased. Upon release from lockdown and return to kindergarten, the familiarity of the school environment, activity classes, and outdoor areas largely motivated the children’s PA behavior, resulting in a compensatory increase in their PA levels, which manifested as higher PA levels compared with those before lockdown. The results of this study showed a significant “supercompensation” effect for MVPA levels, but not for LPA. This may be related to the fact that MVPA is more dependent on nursery conditions and the outdoor environment, whereas LPA is less restricted by such environments [34, 35].

### Psychological

This study found that the SDQ and various dimension scores were better after lockdown than before lockdown, except the pro-social behavior and behavioral problems dimensions, with a significant difference between the total score and pro-sociality (*p* < 0.05). This suggests that a “supercompensation” effect on mental health may have occurred after lockdown. The SDQ scale has been used to study the mental health of young children and preschoolers during lockdown [13, 14], but no studies have compared the scores before and after lockdown.

Research suggest that the human body may experience potentially positive effects after a major public event or disaster due to psychological resilience [36]. In this study, a “supercompensation” effect of psychological resilience was observed in preschoolers after the lockdown, which may be related to parental and environmental factors. On the other hand, the increased time spent with parents during lockdown may have a positive effect on children’s psychological well-being [37, 38], and parents’ attention to their children does not decrease significantly after lockdown due to concerns about the uncertainty of the COVID-19 [39]. The temporary closure of kindergartens during the lockdown period may have reduced children’s opportunities to interact with their peers and teachers [31]. This may have a negative effect on their psychological well-being, which may change from negative to positive when they are re-introduced to familiar peers and teachers after the lockdown is lifted. The combination of these two effects leads to a “supercompensation” effect on children’s mental health following lockdown. The results of this study show a significant decrease in pro-social behavior, which may be related to the slower recovery of this dimension compared with other dimensions, suggesting that families and kindergartens should continue to pay attention to pro-social behavior and strengthen guidance and interventions for this behavior in children.

### Sleep

In this study, the total CSHQ and the various dimension scores were better after lockdown than before lockdown, with a highly significant difference between the total scores and the morning rising dimension score (*p* < 0.01). Further, the detection rate of normal sleep (40.35%) was significantly higher after lockdown than before lockdown (14.04%%) (*p* < 0.01). This suggests that a “supercompensation” effect on sleep may have occurred after lockdown. One study found that sleep indicators deteriorated significantly (*p* < 0.01) during lockdown compared with that before lockdown [40]. In conjunction with this research, a similar “baseline-inhibition-supercompensation” process of PA changes may occur in preschoolers’ sleep habits before, during, and after lockdown.

This study suggests that the ‘baseline-inhibition-supercompensation” changes in the sleep habits of children before, during, and after lockdown are related to changes in their PA and psychology. As described above, both PA and psychological “supercompensation” effects may have occurred in children after lockdown. Many studies have found a significant correlation between sleep and PA in children [31–44]. Inhibition and supercompensation changes in PA in children before, during, and after lockdown can affect their sleep behavior in the respective phases, resulting in similar trends. Similarly, one study found that all dimensions of SDQ mental health indicators significantly correlated with the total CSHQ scores during the lockdown period of the epidemic (*p* < 0.001) [40], the study concluded that sleep problems were associated with emotional, behavioral, hyperactivity, and peer interaction problems during lockdown. After desegregation, the sleep problems of the children were compensated for by the “supercompensation” effect of mental health.

### Limitations

Despite the difficulties of the epidemic, the study was completed by collecting indicators as objectively and comprehensively as possible, but the following shortcomings were observed. Some children did not return to their kindergartens to resume normal studies after the lockdown, which resulted in the inability to collect post-lockdown data and a large sample loss. Furthermore, although the team was approved to be stationed in the kindergartens to perform the tests, we could not stay for a long period to observe the duration and trend of the “overcompensation” effect on the children.

## Conclusion and outlook

### Conclusion

After lockdown, children’s PA levels, psychological health, and sleep health and their multidimensional indicators were better than those before lockdown, with significant changes in MVPA levels, SDQ total scores, and CSHQ total scores, suggesting that children’s PA, psychological health, and sleep health were supercompensated. The children’s PA may show a “baseline-inhibition-supercompensation” process before, during, and after lockdown, and the children’s sleep pattern may also show a similar trend.

### Outlook

#### Exploring the duration and trends of the “supercompensation” effect

Although the study was approved by the epidemic prevention and control department, the Education Bureau, and kindergarten and all the tests were completed in the kindergarten schools after the lockdown, we could not extend our stay to track the changes in the indicators over a longer period of time. Regarding the “supercompensation” theory in the field of sports training, after a period of supercompensation, the body returns to its original level [17, 18]. This assumption holds true in the field of exercise training. The duration of the “supercompensation” effect and the factors influencing it, as well as whether it returns to the pre-lockdown level, need further investigation.

#### Exploring the causes and mechanisms of the ‘supercompensation” effect

Many factors, including family, environment, and policy, influence PA, psychology, and sleep in preschoolers. The factors that are critical to the “supercompensation” effect of lockdown in preschoolers and how they interact with each other are unclear. In addition, a strong association exists between PA, psychological, and sleep indicators in preschoolers, and one of these indicators may play a mediating role. Furthermore, the physiological role of the various body systems, organs, tissues, and cells in the development of the “supercompensation” effect requires further investigation. Such studies will be important to examine the mechanism of the “supercompensation” effect and for the development of subsequent intervention studies.

#### Exploring the “supercompensation” effect for a wider population

Compared with preschoolers, adults, especially the middle-aged and elderly, and other special groups suffering from diseases, may have more prominent PA, psychological, and sleep problems, and the factors affecting various health indicators during lockdown may be more complex. Therefore, future studies should be conducted on a wider range of populations to provide basis for the improvement of isolation measures and implementation of health promotion for multiple populations in the post-epidemic era and to contribute to the safety of life and health for all.

#### Exploring the “supercompensation” effect of major public events

The present study explored the “supercompensation” effect of lockdown for preschoolers during the epidemic, which not only provides basis for the improvement of home isolation measures and implementation of health promotion for preschoolers in the post-epidemic era, but also provides some insights into home isolation after other major public events. Nevertheless, significant differences exist in time, space, intensity, and influencing factors between home isolation during public events such as typhoons, natural disasters, and climate change and lockdown during epidemics. Future research needs to be tailored to the actual situation and examine the effects of “supercompensation” for each type of major public event.

## Data Availability

The datasets used and analyzed during the current study are available from the corresponding author on reasonable request. The materials used in this article are open access or allowed.
